# Soil data from the Barbastro-Balaguer gypsum belt, NE Spain

**DOI:** 10.1016/j.dib.2024.111236

**Published:** 2024-12-17

**Authors:** Juan Herrero, María Tierra, Carmen Castañeda

**Affiliations:** Estación Experimental de Aula Dei, EEAD - CSIC, Ave. Montañana 1005, 50059 Zaragoza, Spain

**Keywords:** Environmental protection, Gypseous soils, Micromorphology, Natura 2000 Network

## Abstract

The dataset [1] hosts pedological info and images of the lands —locally known as *chesas*— of the outcropping gypsiferous core of the Barbastro-Balaguer anticline (Fig. 1). It stands out in the landscape for the linear reliefs due to outcrops of dipping strata with differential resistance to erosion, and also because of its whitish color (Fig. 2) and gypsophilous vegetation. This gypsum outcrop was named in the 19^th^ Century [2] as a gypseous belt, and has been further studied by other geologists like [3,4] and by civil engineers e.g. Hué and Llamas [5]. Traditionally chesas were rangeland, with sparse almond and olive trees and rainfed winter cereals confined at the flat —and often terraced— valley bottoms, or *vales* as known in NE Spain. The chesas have attracted the attention of botanists [[6], [7], [8]], foresters [9,10], and soil hydrophysical properties researchers [11]. Moreover, public interest is increasing as the administrations are establishing rules for nature protection in the gypseous lands, e.g., a demarcation of 137 km^2^ set within the chesas was declared a Special Conservation Area “ES2410074 Yesos de Barbastro”, and then protected by the Habitats Directive of European Union. Also, plant physiologists are focusing on the adaptations of plants to gypsum as reviewed by Escudero et al. [12]. No soil map is available, but according to [13,14] the Gypsic Haploxerepts [15] are dominant. In the absence of a soil map, our dataset can help in the decisions to be made by the authorities, as is the case for water allocation to irrigated estates both in operation and planned, or for authorizations for the spreading of pig slurry.

The herein presented soil data were collected with the classical techniques of pedological prospection. The dataset [1] contains the scans in .TIFF format of 150 whole thin sections of the soils, under both plane polarized light (PPL) and cross polarized light (XPL). Moreover, this dataset directs to a freely downloadable book [16] with the corresponding pedological descriptions, chemical and physical analyses, hydrophysical data, and scanning electron microscope images of the soils, plus micrographs of relevant pedofeatures of thin sections seen under petrographic microscope. The dataset [1] also presents a .xlsx file with an English translation of all figure captions of [16], including those of micrographs, and two more .xlsx files with analytical data. All data can be reused directly by naturalists, engineers, technicians and public servants in charge of environmental law development and enforcement, as well as by people involved in citizen science activities. Thin sections remain stored at EEAD and can be examined at our premises upon request.

Specifications Table

**Table:** 

Subject	Soil Science
Specific subject area	Micromorphology and composition of gypseous soils.
Type of data	TIFF Images, Excel Tables
Data collection	Stereoscopic photointerpretation, field reconnaissance of landforms and hand-auger holes were performed at the beginning. Soil profiles description and sampling were conducted in pits opened with backhoe. Moreover, we carved undisturbed soil blocks and manufactured thin sections for their study under polarizing microscope.
Data source location	The soil samples were collected in the following municipalities in NE Spain: Peraltilla, Almunia de San Juan, San Esteban de Litera, Tamarite de Litera, Alcampell, Ivars de Noguera, Camarasa, Ivorra, and Torá. The location of the respective sampling areas is shown in Fig. 1. The glass-mounted thin sections of the soils are stored at EEAD-CSIC, Zaragoza, Spain.
Data accessibility	Repository name: Mendeley Data; DOI: 10.17632/r57drh58pm.4

A very typical geoform in the gypseous areas of NE Spain are the *vales*, plural form of *val.* The bottoms are often cultivated while the fine materials from the slopes are easily washed away. Fig. 2 shows three examples of vales.

**Fig. 1:**
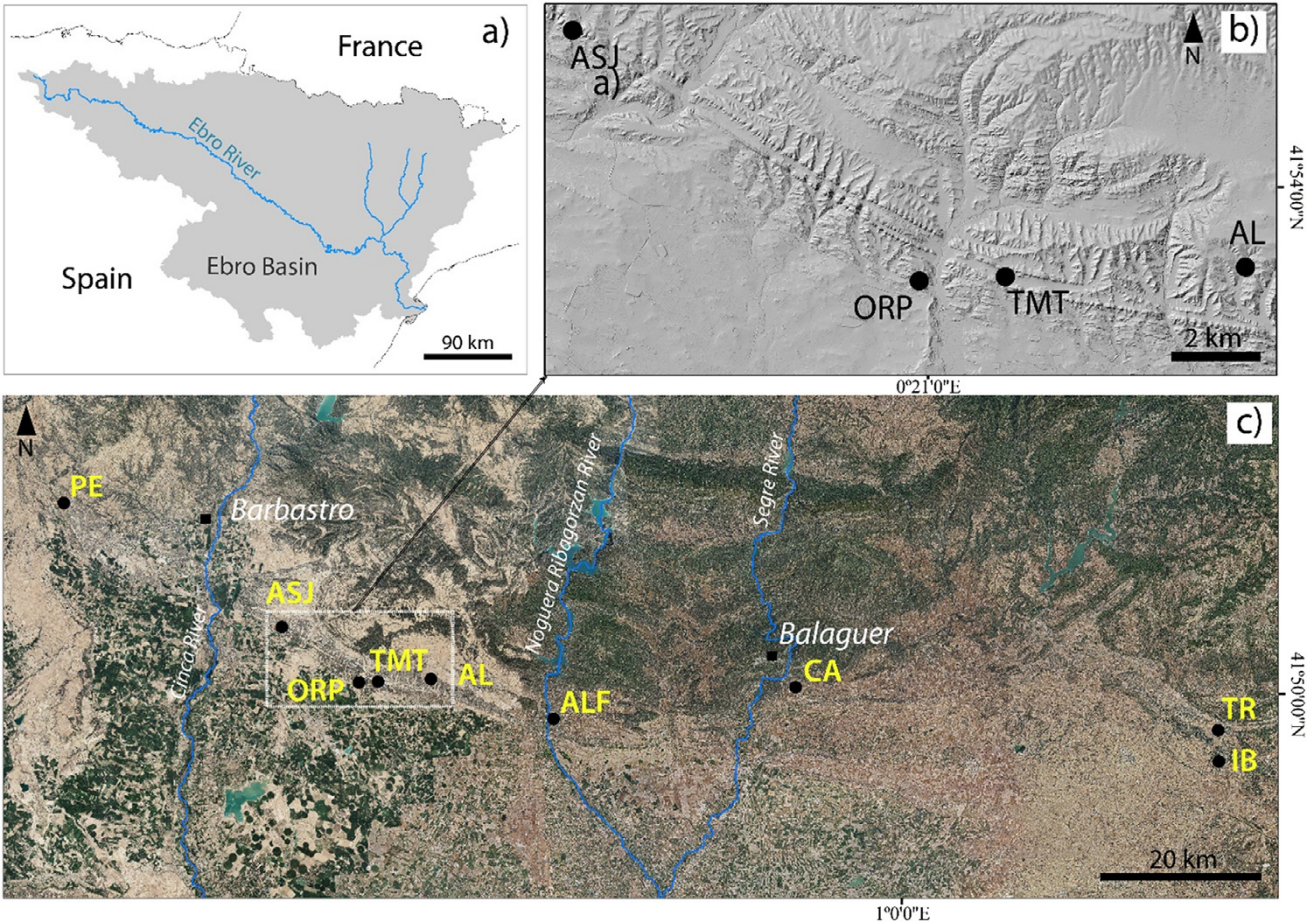
a) The Ebro Basin location in Spain with the Ebro river and the main tributaries that cross the Barbastro-Balaguer anticline outcrop; b) Shaded relief from LiDAR-PNOA-cob2 2015 CC-BY 4.0 scne.es of a stretch of the anticline showing the characteristic parallel alignments of reliefs; c) location of the nine sampling areas on an Ortho-PNOA 2021 CC-BY 4.0 scne.es.

**Fig. 2:**
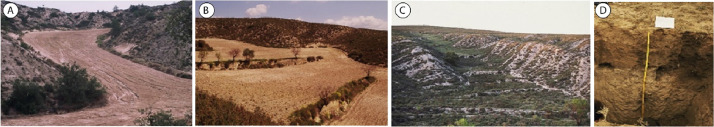
A) The non-terraced bottom of this val is plowed for the next sowing of winter cereal. Rill erosion in the field is evident in spite of the lateral trenches and the berms built with gypseous materials from the slopes. B) A terraced val. Some stunted almond and/or olive trees, vegetate on the transversal man-made ridges. C) This val was part of a military camp, vegetation covers the bottom and its environs; D) Profile in an agricultural terrace. The white gypseous horizon goes from 60 cm deep to the pit bottom of about 160 cm-deep. During > 40 years previous to the picture the cultivation was interrupted, undergoing only sheep grazing.

## 1 Value of the Data

• To our knowledge, the data set is the most complete —if not the only one— for chesa soils, and will be very useful for undertaking soil mapping.
• Natural science researchers will reuse the images and other information when comparing or confirming data acquired elsewhere on gypsum-rich soils. The dataset allows browsing through the images to plan the examination of thin sections in our lab.
• The micrographs provide information about the soil microstructure critical for understanding the plant-soil interface and the soil hydric behavior.
• The data will help to assess the feasibility of forestry or agricultural developments, such as reforestations or planned irrigation schemes, and to monitor the effects of any land transformation.
• Our data can help to evaluate the effects of pig slurry sprawl from the thriving number of intensive swine farms.
• These data will help Spanish and European authorities to attune environmental and agricultural policies to gypseous soils.

## 2 Background

The world's scientific literature has paid little attention to gypseous soils because of their limited extent in temperate-humid countries and their marginal value for agriculture —even if not saline— due to their poor water and nutrient holding capacity. In addition, gypsum dissolution produces surface karst features and subsurface voids which lead to land subsidence and to corrosion of iron and concrete by sulfated waters. These facts pose problems for the field works necessary for the proper functioning of irrigation devices and infrastructures, and for civil engineering in general.

Within this framework, a knowledge gap was detected for soils developed on the Barbastro-Balaguer gypsum belt. As a contribution to fill this gap, we undertook a field reconnaissance followed by hand auger drilling and by the opening of pits for soil description, sampling, classical lab analyses [16], and clay mineralogy determinations using X-ray diffraction [17]. Clods from different soil horizons were collected for scanning electron microscope examination. Unaltered blocks of soil were carved, and thin sections manufactured for observation under petrographic microscope. The presented dataset gives access to scans of soil thin sections and other original information on chesa soils produced so far by the authors.

## 3 Data Description

The dataset [1] is composed by a zip file plus three excel files: (i) the file *Chesas_150thinsections.zip* contains the 300 scanned images under both PPL and XPL of 150 soil thin sections of about 25 µm thick stored at EEAD-CSIC; (ii) the file *Chesas_Fig.captions1991-01.xlsx* shows the translation into English of the captions of the 159 figures in our Spanish book [16] i.e., microphotographs of the soil thin sections plus other figures like sketch maps, geomorphic diagrams, field pictures, and electronic microscope photographs; (iii) the file *ChesasGyps_Peraltilla-Tora-Iborra.xlsx* shows the analytical data of 111 soil samples mentioned in [16], and (iv) the file *ChesasGyps_Tamarite.xlsx* contains compositional data for the 33 soil samples taken in Farrachuela, a 3.4-hectare intermittently flooded hypersaline wetland located in the chesas and marked as TMT in Fig. 1b and c. This Figure also shows the geographic distribution of the soil sampling areas, listed in Table 1.

**Table 1:** Codes naming the scans of thin sections hosted in [1], showing the acronym of the sampling areas and the soil profile identifiers marked in the soil thin sections, plus the name of the municipality.

Acronym	Soil profile identifier in the thin sections	Municipality
PE	11, 13, 14, 15, 18, 19, 20, 21, 22, 28, 29, 34.	Peraltilla
ASJ	ASJ3.	Almunia de San Juan
ORP	ORP.	San Esteban de Litera
TMT	TMT-2, TMT-2bis.	Tamarite de Litera
AL	205.	Alcampell
ALF	41, 42, 43, 44, 45, 47, 48, 49.	Ivars de Noguera
CA	161, 162, 163, 164, 166.	Camarasa
IB	134, 135, 136, 137, 138, 140, 141, 142, 143, 144, 145, 146, 151, 153.	Ivorra
TR	147, 152.	Torá
L	L_8, L_9, L8_L9, SUR_L6.	Several municipalities

Table 2, Table 3, Table 4 display the number and type of analyses conducted in the soil samples presented in the files *ChesasGyps_Peraltilla-Tora-Iborra.xlsx* and *ChesasGyps_Tamarite.xlsx* [1]. The analytical methods are detailed in [16].

**Table 2:** Number and type of the 647 lab analyses performed for characterization of the soil samples presented in the files *ChesasGyps_Peraltilla-Tora-Iborra.xlsx* and *ChesasGyps_Tamarite.xlsx*.

Profile identifier	pH soil:water 1:2.5	Organic matter, %	Calcium carbonate equivalent, %	Gypsum, %	Electrical Conductivity 1:5, dS m^-1^ 25°C
IB-134	4	4	4	4	4
IB-135	4	4	4	4	4
IB-136	1	1	1	1	1
IB-137	3	3	3	3	3
IB-138	4	4	4	4	4
IB-140	6	6	6	5	5
IB-141	4	4	4	4	4
IB-142	4	4	4	4	4
IB-143	3	3	2	3	3
IB-144	6	6	4	5	5
IB-146	4	4	4	4	4
IB-151	3	3	3	2	3
IB-153	2	2	6	2	6
PE-11	6	6	6	6	6
PE-14	14	14	14	14	7
PE-15	6	6	6	6	6
PE-20	5	5	5	5	5
PE-22	4	4	4	4	4
PE-28	3	3	3	3	3
PE-29	5	5	5	5	5
PE-34	3	3	3	3	3
TMT-1	6	0	6	6	6
TMT-2	6	0	6	6	6
TMT-3	6	0	6	6	6
TMT-4	6	0	6	6	6
TMT-5	6	0	6	6	6
TMT-6	1	0	1	1	1
TR-147	7	7	7	6	6
TR-152	7	6	7	7	0

**Table 3:** Number and type of the 603 salinity analyses performed in the soil samples presented in the files *ChesasGyps_Peraltilla-Tora-Iborra.xlsx* and *ChesasGyps_Tamarite.xlsx*.

Profile identifier	SP^1^	ECe^1^, dS m^-^1 25°C	Ca^2^^+^	Mg^2^^+^	Na^+^	CO_3_^2^^+^	HCO_3_^–^	SO_4_^2^^+^	Cl^-^	SAR^3^	pHe 4
IB-135	4	4	4	4	4	0	0	0	0	0	0
IB-136	1	1	1	1	1	1	1	1	1	1	0
IB-137	3	3	3	3	3	3	3	3	3	3	0
IB-138	4	4	4	4	4	4	4	4	4	4	0
IB-142	3	3	3	3	3	3	3	3	3	3	0
IB-151	3	3	3	3	3	0	0	0	0	0	0
IB-153	2	2	2	2	2	2	2	2	2	2	0
PE-11	6	6	6	6	6	6	6	6	6	6	0
PE-14	5	5	5	5	5	5	5	5	5	5	0
PE-22	4	4	4	4	4	4	4	4	4	4	0
PE-29	5	5	5	5	5	5	5	5	5	5	0
TMT-2	8	8	8	8	8	8	8	8	8	8	8
TMT-3	8	8	8	8	8	8	8	8	8	8	8
TMT-6	1	1	1	1	1	1	1	1	1	1	1
TR-147	6	5	5	5	5	5	5	5	5	5	0

1 Saturation percentage.

2 Electrical conductivity of the saturated paste.

3 Sodium Adsorption Ratio.

4 pH in the saturation extract.

**Table 4:** Number and type of the 144 analyses of particle size distribution, exchangeable cations, and cation exchange capacity in the soil samples presented in the files *ChesasGyps_Peraltilla-Tora-Iborra.xlsx* and *ChesasGyps_Tamarite.xlsx*.

Profile identifier	PSD^1^	Exchangeable cations				CEC^2^
		Ca^2^^+^	Mg^2^^+^	Na^+^	K^+^	
IB-134	2	1	2	2	2	1
IB-135	3	3	3	3	3	3
IB-136	1	1	1	1	1	1
IB-137	1	0	0	0	0	0
IB-138	4	4	4	4	4	4
IB-140	3	4	4	4	4	4
IB-141	3	3	3	3	3	3
IB-142	3	0	0	0	0	0
IB-143	2	0	0	0	0	0
IB-144	3	0	0	0	0	0
IB-146	3	0	0	0	0	0
IB-151	1	0	0	0	0	0
TR-147	2	6	6	6	6	6

1 Particle Size Distribution.

2 Cation Exchange Capacity.

## 4 Experimental Design, Materials and Methods

Undisturbed soil blocks, specimens of gypsum rock, and lichens with their gypseous substrate were impregnated with a cold-setting polyester resin. Then, thin sections were manufactured as per Guilloré [18]. The glass holders were imprinted with the acronym for the corresponding municipality followed by the numerical identifier of the soil profile (Table 1) and with the upper and lower depth of the unaltered soil block. Each of the 150 whole thin section holders was scanned with a resolution of 1200 dots per inch under both PPL and XPL. The name of the scans ends with “_001” for PPL, and with “_003” for XPL. Most of the glass holders have an arrow pointing upwards and/or a notch with the same meaning.

Remarkable microscopic pedofeatures pertaining to the scanned thin sections are illustrated in the microphotos contained in [16]. Some of them are polarizing microscope images of the ubiquitous gypsum that appears as: (i) crystals up to several mm in size and often dispersed in a silicatic-carbonatic groundmass, and (ii) monomineral masses of microlenticular gypsum crystals typically sized < 15 µm; under PPL, these crystals, randomly overlapped in the thin section, produce a yellowish faint color in plain light which intensifies as the microscope diaphragm is closed, similarly to that described by Stoops and Ilaiwi [19] in Syria. Other outstanding pedofeatures include the celestite crystals —up to 60 µm long— and their nests, or the intriguing millimetric complex feature combining biogenic calcification crystals and decalcification domains. This pedofeature —named quera [13]— has also been found in loess and other materials, as reviewed by [20].

## Limitations

The number of particle size distribution analyses in Table 4 has been simplified by no expressing the results for the four separates (coarse sand, sand, silt, clay). The reason is that in many cases the separation was either impossible or not fully achieved, at least for the finer fractions, due to the classical phenomena of flocculation when dispersing gypsum rich materials. Moreover, the reservations related to the drawbacks of particle size distribution determinations in gypsiferous materials must be considered.

The 128-thickness data of the sampled soil layers —horizons or other— provided in [1] allow to calculate total and average contents for groups of layers, but such calculations would not make sense for non-additive magnitudes like ECe, saturation percentage, pH, or SAR.

## Ethics Statement

The authors have read and follow the ethical requirements for publication in Data in Brief and confirm that the current work does not involve human subjects, animal experiments, or any data collected from social media platforms.

## Credit Author Statement

**Juan Herrero:** Conceptualization, Methodology, Writing original draft. **María Tierra:** Data curation, Visualization. **Carmen Castañeda:** Visualization, Reviewing and Editing, Funding acquisition. All authors have read and approved the final version.

## Data Availability

Mendeley DataGypsum from soils of chesas, NE Spain (Original data).

## References

[1] Herrero J., Tierra M., Medina E.T., Castañeda C. (2024). Gypsum from soils of chesas, NE Spain. Mendeley Data.

[2] Mallada L. (1878). Pages. Facsimile edition ISBN: 978-84-86856-40-3.

[3] Pardo G., Villena J. (1979). Aportación a la geología de la región de Barbastro. Acta Geolog. Hispanica.

[4] Lucha P., Gutiérrez F., Galve J.P., Guerrero J. (2012). Geomorphic and stratigraphic evidence of incision-induced halokinetic uplift and dissolution subsidence in transverse drainages crossing the evaporite-cored Barbastro-Balaguer Anticline (Ebro Basin, NE Spain). Geomorphology.

[5] Hué F., Llamas M.R. (1960).

[6] Ferrández-Palacio J.V., Palomares A., Rovira J. (2008). (Coords.) Comarca de la Litera.

[7] Martínez-Hernández F., Medina-Cazorla J.M., Mendoza-Fernández A., Pérez-García F.J., Sánchez-Gómez P., Garrido-Becerra J.A., Gil C., Mota J.F. (2009). Preliminary essay on the chorology of the Iberian gypsicolous flora: rarity and richness of the gypsum outcrops. Acta Bot. Gallica.

[8] Blanco-Sánchez M., Moore M.J., Ramos-Muñoz M., Pías B., García-Fernández A., Prieto M., Plaza L., Isabel I., Escudero A., Matesanz S. (2021). Phylogeography of a gypsum endemic plant across its entire distribution range in the western Mediterranean. Am. J. Bot..

[9] Olarieta J.R., Usón A., Rodríguez R., Rosa M., Blanco R., Antúnez M. (2000). Land requirements for *Pinus halepensis* Mill. growth in a plantation in Huesca, Spain. Soil Use Manage..

[10] Olarieta J.R., Rodríguez-Ochoa R., Ascaso E. (2012). Soil gypsum and increased penetration resistance restrict early growth of *Quercus ilex* plantations. Arid Land Res. Manage..

[11] Moret-Fernández D., Herrero J. (2015). Effect of gypsum content on soil water retention. J. Hydrol..

[12] Escudero A., Palacio S., Maestre F.T., Luzuriaga A.L. (2015). Plant life on gypsum: a review of its multiple facets. Biol. Rev..

[13] Artieda O., Herrero J. (2003). Pedogenesis in lutitic Cr horizons of gypsiferous soils. Soil Sci. Soc. Am. J..

[14] Badía D. (2009). Itinerarios edáficos por el Alto Aragón.

[15] Soil Survey Staff (2022).

[16] Herrero J. (1991). http://hdl.handle.net/10261/84695.

[17] Herrero J., Jiménez-Ballesta R., Castañeda C. (2024). The clay minerals in the soils of the gypseous belt of Barbastro, NE Spain. Land.

[18] Guilloré P. (1985). Département des Sols.

[19] Stoops G., Ilaiwi M. (1981). International Soil Classification Workshop, April 1980.

[20] Álvarez A., Torres-Guerrero C.A., Travé A., Preusser F., Plata J.M., Poch R.M. (2024). Biogenic carbonates (*queras*) in loess-palaeosol sequences of the Ebro Basin and their potential use as a palaeoenvironmental proxy. Catena.

